# Late Dislodgement of a Leadless Pacemaker: Potential Role of Impedance Decline as an Early Warning Sign

**DOI:** 10.1002/joa3.70187

**Published:** 2025-09-09

**Authors:** Toshihiko Goto, Masashi Yokoi, Kento Mori, Yoshihiro Seo

**Affiliations:** ^1^ Department of Cardiology Nagoya City University Graduate School of Medical Sciences Nagoya Japan

**Keywords:** dislodgement, impedance monitoring, leadless pacemaker, pacemaker extraction

## Abstract

Pacing impedance declined rapidly 1 week after implantation, followed by a significant increase in threshold at the 2‐week follow‐up, resulting in pacing failure.

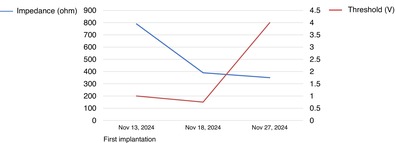

An 88‐year‐old man was hospitalized with immune‐mediated neuropathy. Despite intravenous immunoglobulin therapy, his muscle weakness persisted and worsened 1 month after admission. One week after this deterioration, he experienced a 10‐s cardiac arrest due to a paroxysmal atrioventricular block at night. No reversible causes were identified, such as electrolyte abnormalities or medications, and the left ventricular function was preserved. Although the patient was asymptomatic because he was in bed at that time, a shared decision was made to implant a leadless pacemaker as a backup. Given that the pacing indication was for backup only, we selected the Aveir VR (Abbott, Chicago, IL), which was successfully implanted at the apical septum with 1.5 turns, achieving an adequate injury current. Immediately postimplantation, the pacing threshold was 1.0 V at 0.4 ms, impedance was 790 Ω, and R‐wave sensing measured 9.0 mV. One week later, the threshold improved to 0.75 V at 0.4 ms; however, the impedance had declined to 390 Ω (R‐wave sensing: 13.8 mV).

Two weeks postimplantation, the threshold increased to 4.0 V at 0.4 ms, resulting in pacing failure, and the impedance further declined to 350 Ω. Cine angiography revealed that the chevron marker of Aveir was rotated by approximately 1/4 turn compared to the postoperative image, suggesting partial dislodgement (Figure 1A,B). Immediate removal was considered; however, the patient's platelet count decreased from 117 × 10^9^/L at implantation to 14 × 10^9^/L owing to disease progression, increasing the risk of bleeding. At that time, although the patient experienced frequent episodes of atrioventricular block, he showed no symptoms such as syncope or loss of consciousness, likely because he was bedridden due to the progression of his underlying condition. Therefore, conservative management, including platelet transfusions, was selected. At this point, high‐sensitivity cardiac troponin I increased to 112 pg/mL, from 42.2 pg/mL at admission. C‐reactive protein remained unchanged at 0.42 mg/mL at both time points.

**FIGURE 1:**
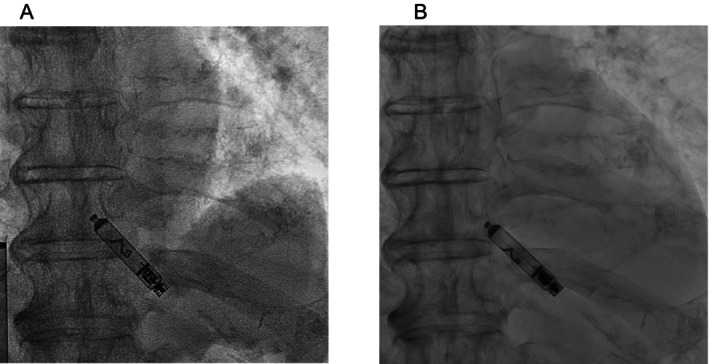
(A) Postoperative cine angiography image and (B) cine angiography image taken 2 weeks after the procedure. Both images are in the right anterior oblique 30° view. In (B), the chevron marker appears to have rotated by approximately 1/4 turn compared to (A).

Six weeks after implantation, routine chest radiography confirmed that the Aveir had migrated outside the heart (Figure 2A). At this time, the patient's platelet count had improved to 64 × 10^9^/L, allowing for transvenous extraction. An 8.5‐F steerable sheath (Agilis NxT, St. Jude Medical, St. Paul, MN) threaded through the 27‐F Aveir introducer was initially used but did not reach the dislodged Aveir and rather hindered its extraction. Instead, a 6‐F Judkins right guiding catheter (Hyperion JR4; Asahi Intec, Aichi, Japan) was inserted directly through the Aveir introducer. A gooseneck snare was used to grasp the helical portion of the dislodged Aveir (Figure 2B,C), successfully retracting it into the right atrium and storing it within the Aveir introducer (Figure 3A,B). Consequently, the Aveir was safely removed from the body. Right ventriculography confirmed that the right ventricle and left pulmonary artery were intact. Considering the patient's 10 kg weight loss from his baseline weight of 50 kg due to prolonged bed rest, decreased frequency of atrioventricular block with clinical improvement, and existing right femoral access for device removal, we opted to implant a new Aveir via the same site instead of switching to a transvenous pacemaker. A new Aveir was implanted in the inferior septum to achieve adequate fixation and an appropriate injury current. Postimplantation measurements showed a pacing threshold of 0.5 V at 0.4 ms, impedance of 660 Ω, and R‐wave sensing of 9.0 mV. Histopathological examination of the helix‐attached tissue revealed blood components, inflammatory cells, and degenerated cells, but no myocardium.

**FIGURE 2:**
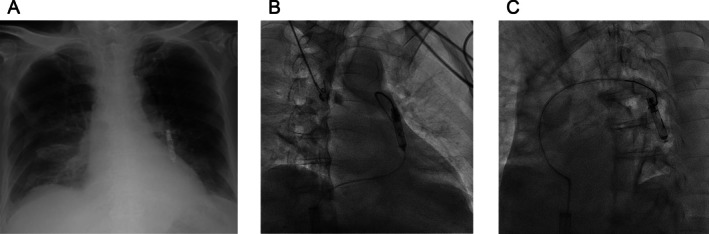
(A) Chest radiograph (posterior–anterior view) showing the dislodged leadless pacemaker located outside the heart. (B) Cine angiography image in the right anterior oblique 30° view, showing the snare catheter capturing the helical portion of the leadless pacemaker. (C) The same maneuver viewed in the left anterior oblique 50° view.

**FIGURE 3:**
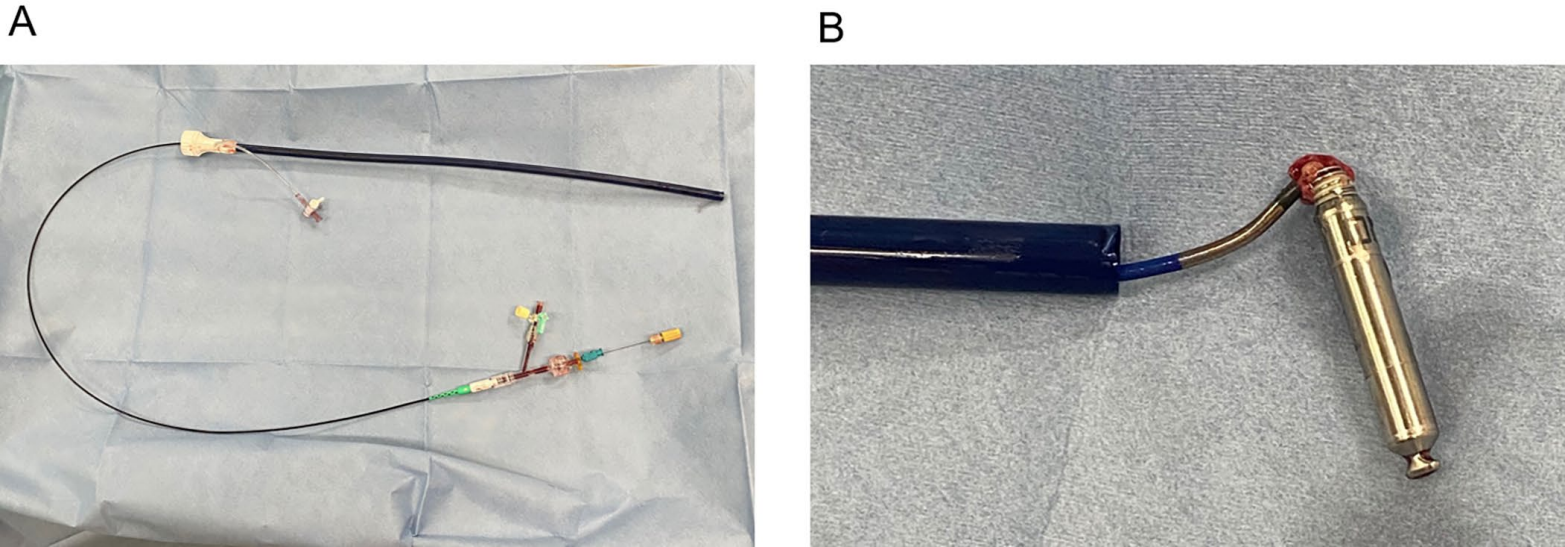
(A) The extraction system, comprising a right guiding catheter inserted through an introducer. A snare catheter passed through this system was used to secure the leadless pacemaker. (B) The extracted leadless pacemaker.

Leadless pacemakers provide an alternative to traditional transvenous pacing systems and reduce complications such as lead fractures, infections, and venous stenosis. However, despite their lower long‐term risk profile, rare complications, such as device dislodgement, remain a concern. Most reported dislodgements occur within 2 weeks postimplantation, often resulting in embolization to the pulmonary artery or right ventricle. However, cases of late dislodgement after the acute phase have rarely been reported. Moreover, no previous reports have highlighted impedance decline as an early predictor of Aveir dislodgement. Here, we present a case of late Aveir dislodgement, underscoring the role of impedance decline as a potential early warning sign.

Two primary mechanisms of leadless pacemaker dislodgement have been proposed: acute dislodgement due to inadequate initial fixation and nonacute dislodgement from mechanical stress or myocardial remodeling [1]. Since dislodgement can only be confirmed after it has occurred, and given the 6‐week interval in our case, it most likely falls into the latter category, the dislodgement process may have begun earlier. In autoimmune diseases, shared antigens between the nervous system and myocardium can trigger myocarditis, as reported in severe cases of Guillain‐Barré syndrome [2]. Although histopathological findings were inconclusive, myocarditis associated with immune‐mediated neuropathy may have been present at the time of the first implantation, with its progression contributing to later dislodgement. Supporting this hypothesis, increased pacing thresholds and more frequent episodes of atrioventricular block were observed during disease progression. Conversely, during the 6 months after the second implantation—when the disease had stabilized—the pacing rate remained below 1%, the threshold improved to 0.5 V at 0.4 ms, and the impedance measured 590 Ω (R‐wave sensing amplitude, 14.7 mV). These findings suggest that the late dislodgement of the Aveir in this case may reflect myocardial remodeling due to autoimmune‐associated myocarditis. Six months after the second implantation, the high‐sensitivity troponin I level, which had previously increased to 112 pg/mL, decreased to 45.3 pg/mL—approximately the same as the level at admission. The C‐reactive protein level at that time was 0.34 mg/dL.

To date, few cases of Aveir extraction have been reported. One case involved dislodgement the day after implantation, successfully extracted using a docking system 8 days later [3]. Another case described intraoperative dislodgement with embolization into the left pulmonary artery, retrieved using a sheath‐and‐snare technique [4]. Our experience suggests that alternative access methods may be considered when standard approaches are unsuccessful. In particular, rigid reliance on steerable sheaths, including Agilis, may not always be beneficial, and a direct approach using a guiding catheter may offer better maneuverability.

A notable finding in this case was the decline in impedance preceding the threshold elevation and eventual Aveir dislodgement. To the best of our knowledge, no prior reports have documented an impedance decline as a precursor to Aveir dislodgement. However, in transvenous leads, an association between lead dislodgement and impedance decline has been well recognized [5]. Therefore, this case suggests that, similar to transvenous leads, there may also be an association between impedance decline and device dislodgement in leadless pacemakers. These findings suggest the potential role of impedance decline as an early warning sign for detecting impending dislodgement in leadless pacemakers. Although early intervention was not feasible in this case due to the patient's condition, recognizing impedance trends in future cases could facilitate timely management and improved patient outcomes.

## 1 Conclusion

This case highlights a rare instance of late Aveir dislodgement and suggests that impedance monitoring may be helpful in identifying early signs of device dislodgment. Careful monitoring of impedance trends may allow earlier recognition and intervention in future cases, potentially improving patient outcomes.

## Disclosure

Clinical Trial Registration: N/A.

Permission to Reproduce Material: N/A.

## Ethics Statement

This case report complies with the ethics and integrity policies of the *Journal of Arrhythmia*.

## Consent

Written informed consent was obtained from the patient for the publication of this case report and accompanying images.

## Conflicts of Interest

The authors declare no conflicts of interest.

## Supporting information


**Data S1:** joa370187‐sup‐0001‐FigureS1.pptx. **Figure S1** shows the time course of impedance (ohms) and pacing threshold (V) following the implantation of an Aveir leadless pacemaker. During the first week after the initial implantation, the impedance declined markedly from 790 Ω to 390 Ω. This was followed by an abrupt rise in pacing threshold, ultimately culminating in device dislodgement. Notably, on December 2 and 5, the pacing threshold exceeded 6.0 V (pulse width: 0.4 ms). In contrast, during the 6 months following the second implantation, both the impedance and the threshold remained stable, with no evidence of device dislodgement.

**Data S2:** joa370187‐sup‐0002‐FigureS2.pptx. **Figure S2** shows examples of intracardiac electrograms recorded immediately after the first Aveir implantation. The y‐axis represents the amplitude (mV), and the x‐axis represents the time (ms). The current of injury is clearly visible.

## Data Availability

Data sharing is not applicable to this article because no data sets were generated or analyzed.
