# Supplementary material for: Late Dislodgement of a Leadless Pacemaker: Potential Role of Impedance Decline as an Early Warning Sign
Source: J Arrhythm. 2025 Sep 9;41(5):e70187. doi: 10.1002/joa3.70187 (PMC12418166; doi:10.1002/joa3.70187)
Supplement: Supplementary file 1 — Data S1: joa370187‐sup‐0001‐FigureS1.pptx. Figure S1 shows the time course of impedance (ohms) and pacing threshold (V) following the implantation of an Aveir leadless pacemaker. During the first week after the initial implantation, the impedance declined markedly from 790 Ω to 390 Ω. This was followed by an abrupt rise in pacing threshold, ultimately culminating in device dislodgement. Notably, on December 2 and 5, the pacing threshold exceeded 6.0 V (pulse width: 0.4 ms). In contrast, during the 6 months following the second implantation, both the impedance and the threshold remained stable, with no evidence of device dislodgement. [file JOA3-41-e70187-s001.pptx]

## Slide 1
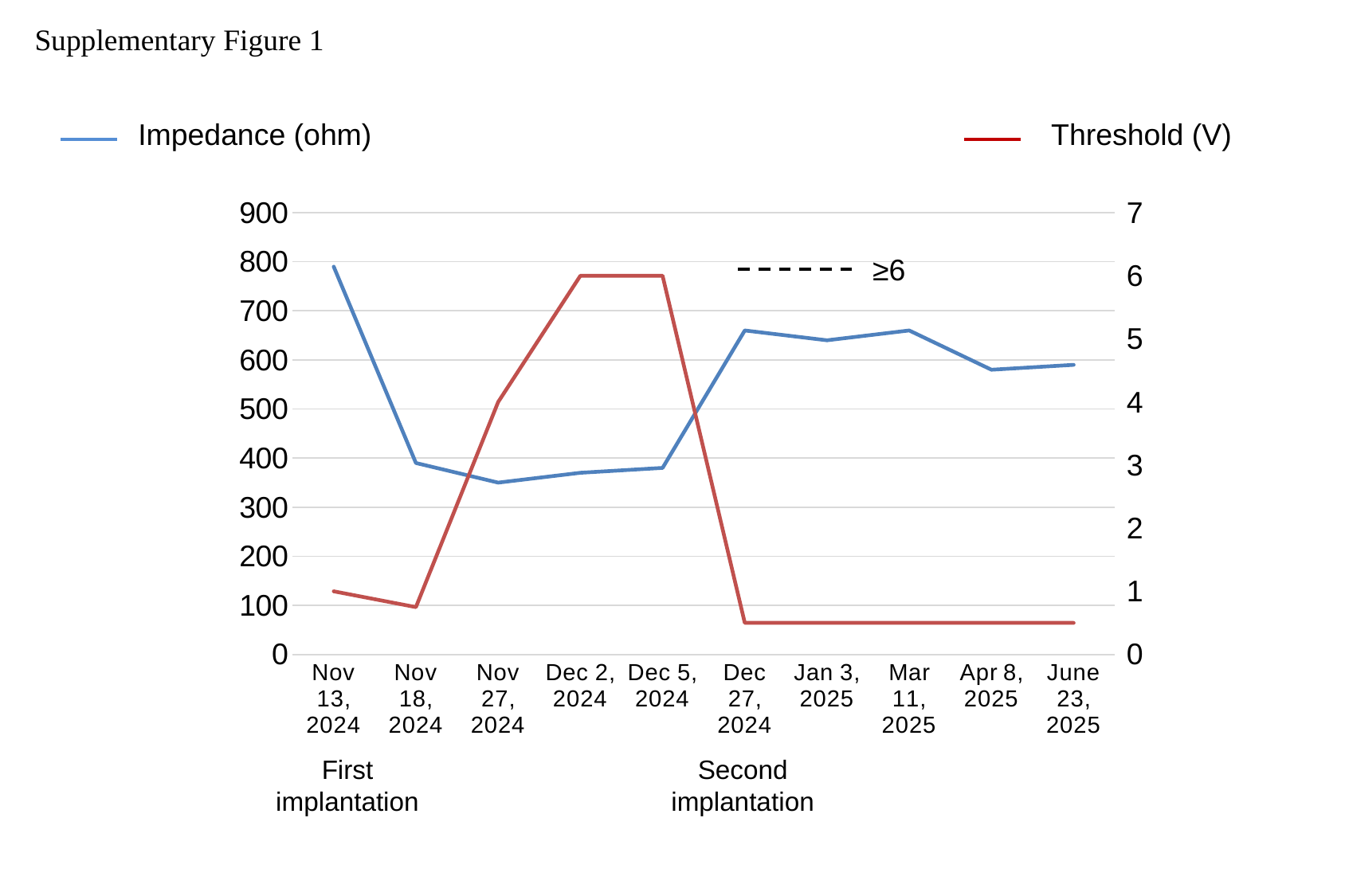

Impedance (ohm)
Supplementary Figure 1
Threshold (V)
R
### Chart
| Category | Impedance | Threshold |
|---|---|---|
| Nov 13, 2024 | 790.0 | 1.0 |
| Nov 18, 2024 | 390.0 | 0.75 |
| Nov 27, 2024 | 350.0 | 4.0 |
| Dec 2, 2024 | 370.0 | 6.0 |
| Dec 5, 2024 | 380.0 | 6.0 |
| Dec 27, 2024 | 660.0 | 0.5 |
| Jan 3, 2025 | 640.0 | 0.5 |
| Mar 11, 2025 | 660.0 | 0.5 |
| Apr 8, 2025 | 580.0 | 0.5 |
| June 23, 2025 | 590.0 | 0.5 |≥6
First implantation
Second implantation
