# Supplementary figures and images for: Late Dislodgement of a Leadless Pacemaker: Potential Role of Impedance Decline as an Early Warning Sign
Source: J Arrhythm. 2025 Sep 9;41(5):e70187. doi: 10.1002/joa3.70187 (PMC12418166; doi:10.1002/joa3.70187)

## Slide 1
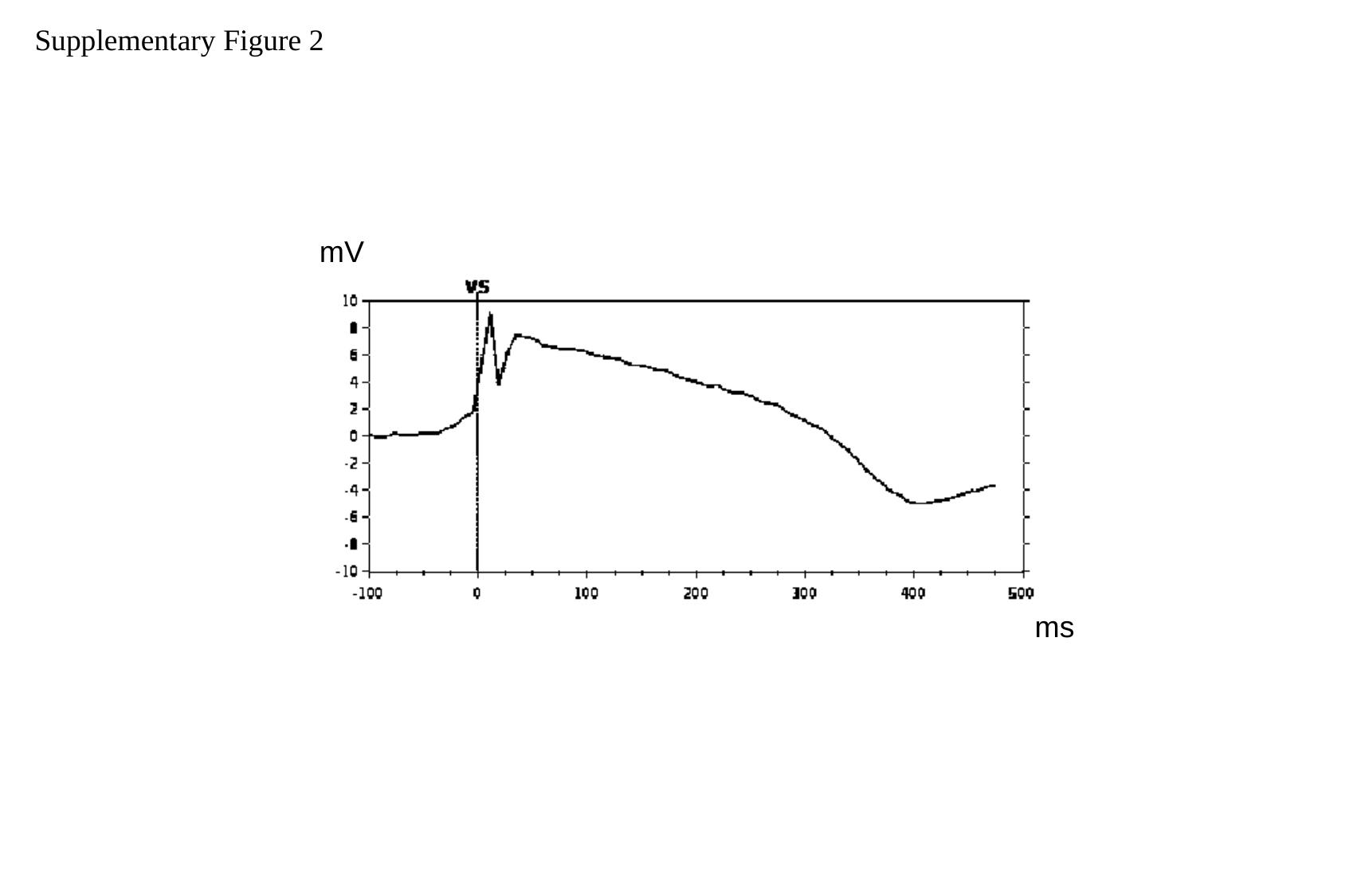

Supplementary Figure 2
mV
ms

Supplement: Supplementary file 2 — Data S2: joa370187‐sup‐0002‐FigureS2.pptx. Figure S2 shows examples of intracardiac electrograms recorded immediately after the first Aveir implantation. The y‐axis represents the amplitude (mV), and the x‐axis represents the time (ms). The current of injury is clearly visible. [file JOA3-41-e70187-s002.pptx]
